# Optimising and Evaluating the Characteristics of a Multiple Antigen ELISA for Detection of *Mycobacterium bovis* Infection in a Badger Vaccine Field Trial

**DOI:** 10.1371/journal.pone.0100139

**Published:** 2014-07-01

**Authors:** Inma Aznar, Klaas Frankena, Simon J. More, Clare Whelan, Wayne Martin, Eamonn Gormley, Leigh A. L. Corner, Denise Murphy, Mart C. M. De Jong

**Affiliations:** 1 Centre for Veterinary Epidemiology and Risk Analysis, School of Veterinary Medicine, University College Dublin, Dublin, Ireland; 2 Quantitative Veterinary Epidemiology group, Wageningen Institute of Animal Sciences, Wageningen University, Wageningen, The Netherlands; 3 Enfer Scientific, Naas, Co. Kildare, Ireland; 4 Professor Emeritus, University of Guelph, Guelph, Ontario, Canada; 5 School of Veterinary Medicine, University College Dublin, Dublin, Ireland; 6 Athlone Regional Veterinary Laboratory, Department of Agriculture, Food and the Marine, Athlone, Ireland; University of Cape Town, South Africa

## Abstract

A long-term research programme has been underway in Ireland to evaluate the usefulness of badger vaccination as part of the national bTB (bovine tuberculosis) control strategy. This culminated in a field trial which commenced in county Kilkenny in 2009 to determine the effects of badger vaccination on *Mycobacterium bovis* transmission in badgers under field conditions. In the present study, we sought to optimise the characteristics of a multiplex chemiluminescent assay for detection of *M. bovis* infection in live badgers. Our goal was to maximise specificity, and therefore statistical power, during evaluation of the badger vaccine trial data. In addition, we also aimed to explore the effects of vaccination on test characteristics. For the test optimisation, we ran a stepwise logistic regression with analytical weights on the converted Relative Light Units (RLU) obtained from testing blood samples from 215 badgers captured as part of culling operations by the national Department of Agriculture, Food and the Marine (DAFM). The optimised test was applied to two other datasets obtained from two captive badger studies (Study 1 and Study 2), and the sensitivity and specificity of the test was attained separately for vaccinated and non-vaccinated badgers. During optimisation, test sensitivity was maximised (30.77%), while retaining specificity at 99.99%. When the optimised test was then applied to the captive badger studies data, we observed that test characteristics did not vary greatly between vaccinated and non-vaccinated badgers. However, a different time lag between infection and a positive test result was observed in vaccinated and non-vaccinated badgers. We propose that the optimized multiplex immunoassay be used to analyse the vaccine trial data. In relation to the difference in the time lag observed for vaccinated and non-vaccinated badgers, we also present a strategy to enable the test to be used during trial evaluation.

## Introduction

Badgers play an important role in the epidemiology of bovine tuberculosis (bTB) in Ireland, by acting as a source of infection to cattle [1], [2]. The prevalence of *Mycobacterium bovis* infection in badgers, based on animals captured as part of culling operations by the national Department of Agriculture, Food and the Marine (DAFM), was estimated recently at 36.3% [3], although this is known to vary substantially between areas where bTB in cattle is problematic [4] or absent [5]. Sustainable progress towards eradication of *M. bovis* infection in cattle might not be possible in the face of continued spillover of infection from badgers to cattle [2].

Several control options are available to limit transmission of infection from badgers to cattle, including reducing the frequency of contact between these species and decreasing the proportion of the badger population susceptible to infection, through vaccination [2]. In Ireland, focused badger culling is being used to reduce contact rates between badgers and cattle in areas of high bTB incidence in cattle. However, it is hoped that badger culling can be replaced by, or supplemented with, badger vaccination. A long-term Irish research programme is on-going to evaluate the usefulness of badger vaccination as part of the national bTB control strategy. A series of pen-based vaccination trials have been conducted, where badgers were vaccinated with Bacillus Calmette-Guerin (BCG) and subsequently challenged with *M. bovis*, and the impact of vaccination on pathology, bacteriology and progression of infection in badgers has previously been reported [6]–[9]. Subsequent to this work, a field trial commenced in county Kilkenny, in 2009, to determine the effects of badger vaccination on *M. bovis* transmission in badgers under field conditions [10]. The field trial design will enable comparison of bTB incidence between vaccinated and unvaccinated badgers in three areas of differing vaccine coverage (100, 50 and 0%).

A number of challenges have been encountered during the design of the field trial in Co. Kilkenny, including: a) the need to fully understand the biology underpinning protection following BCG vaccination, both in individual badgers and within the broader badger population (including the likelihood of reduction of infectiousness and therefore transmission); b) the need to identify the infection status of each badger at each capture event (a capture/recapture design has been employed), and c) the need for sufficient statistical power in the aforementioned design. There is now a better understanding of options to address the first and third of these challenges [10], [11]. In this paper, we consider the second of these challenges, that is, the need for a test to identify the infection status of individual badgers at each capture event. It has been shown recently that this diagnostic test will need a specificity very close to 100% in order to obtain sufficient study power [11]. The authors estimated a minimum specificity of 99.8% to achieve a power above 60% in this trial. The need for a high specificity reflects the fact that the cost of false positive test results is much higher than that of false negative results.

A significant amount of work in relation to diagnostic methods for tuberculosis in live badgers has been conducted in Ireland and the United Kingdom [12]–[17]. Assays based on the measurement of cellular responses, such as gamma-interferon, have attracted considerable interest as they are expected to deliver a higher sensitivity in comparison to antibody-based assays [13], [15]. Furthermore, these cell response assays have the advantage of being able to detect earlier stages of infection [18]. However, one of the drawbacks of these assays is the large effect of pre-culture holding time and temperature on gamma-interferon responses [19], [20]. Several other bTB assays have been developed. The Brock test is an indirect ELISA that measures *M. bovis*-specific antibody responses to a single antigen, MPB83 [21], [22]. MPB83 is expressed by other members of M. tuberculosis complex, but is serodominant in *M.bovis* infection. Subsequent studies have shown that test sensitivity and specificity can be enhanced by using a mixture of antigens rather than a single antigen. Based on the use of a multi-antigen print immunoassay (MAPIA) and culture as the gold standard, the sensitivity was found to increase from 47.4% to 52.6% and the specificity from 89% to 95% [23]. In an attempt to simplify the procedure, thereby allowing badger testing to be performed in the field, a lateral flow immunoassay (the Brock TB Stat-Pak assay; Chembio Diagnostic Systems, Inc., Medford, NY) was developed [14], [16].

Here, we will look at the Enfer chemiluminescent multiplex ELISA system, originally developed for testing *M. bovis* in cattle [24], [25]. The test was adapted for badgers and applied to 200 blood samples from badgers captured in Ireland in areas of high bovine tuberculosis prevalence [26], this study reported a sensitivity and specificity in badgers of 56.7% and 96.99%, respectively, when using a panel of *M. bovis* antigens. The availability of this test, the fact that this test can be performed in stored blood samples without losing sensitivity or specificity, and its quantitative nature, made this test the test of choice. Given this background, the current study had two objectives. First, we sought to statistically optimise a multiplex chemiluminescent assay for detection of *M. bovis* infection in live badgers to maximise specificity, and therefore statistical power, during evaluation of the badger vaccine trial in Ireland. Second, we aimed to explore the effects of vaccination on test characteristics and to review the implications for analysis of the data from the Kilkenny badger vaccine trial.

## Materials and Methods

The 215 blood samples used for test optimization were part of an archive. The badgers from which these samples had been taken, had been captured as part of the DAFM culling operations carried out in the Republic of Ireland. The Department of Arts, Heritage and the Gaeltacht, specifically the National Parks and Wildlife Service, issues licences to the Department of Agriculture, Food and the Marine to undertake the capturing programme. The captive badger studies were carried out under license (No. B100/3187, Cruelty to Animals Act 1876) issued by the Department of Health and Children, and ethical approval was obtained from the UCD Animal Research Ethics Committee (AREC-P-04-28 and AREC-P-24-06).

### 1. Test optimisation using naturally infected wild badgers

#### 1.1 Samples

Serum samples from 215 badgers captured as part of the DAFM culling operations carried out in the Republic of Ireland were used for optimisation of the diagnostic test. Focused (reactive) badger removal is conducted under license in the vicinity of herds that have had a severe bTB breakdown where the cause cannot be attributed to non-wildlife sources. The serum samples had been collected for archiving purposes. A study by Murphy et al. (2010) [3] looked at these badgers for gross visible lesions of TB at post mortem and samples were collected from a range of tissues and pooled into groups for bacterial culture of *M. bovis*. An aseptic technique was used when preparing tissue samples to minimize contamination before culture. In the current study, culture results were used as the gold standard and a badger was considered infected when *M. bovis* was isolated from any of the samples taken.

#### 1.2 Multiplex immunoassay

Antibody responses, expressed as relative light units (RLU), to a panel of antigens were measured using the Enfer chemiluminescent multiplex ELISA system (Enfer Scientific, Co. Kildare, Ireland). The antigen panel consisted of the following six recombinant proteins: MPB83, MPB70, Rv3616c fragment and full protein, ESAT-6 and CFP10, as well as purified protein derivative from *M. bovis* (PPDb) and a peptide of MPB70. Tests were carried out by Enfer Scientific using 96-well microtitreplates. Recombinant antigens (Fusion Antibodies Ltd. (Belfast)) and peptides (Genosphere Biotechnologies (France)) were prepared as previously described by Whelan et al. (2008) [24].

The multiplex assay was carried according to Whelan et al. (2008) [24]. Serum samples were diluted 1∶450 into sample dilution buffer and mixed. A 50 µl sample dilution was added per well. The plates were incubated at room temperature with agitation for 90 minutes. The plates were washed once with Enfer Wash Buffer (Enfer Scientific) and aspirated. The detection antibody (CF2/HRPo Anti-Badger IgG-HRP conjugate, kindly provided by Mark Chambers, AHVLA, Weybridge, UK) was prepared to a dilution of 1∶75,000 in detection antibody dilution buffer. After addition of 50 µl of the detection antibody to each test well, the plates were incubated at room temperature for 30 minutes with agitation. The plates were washed as above and 50 µl of chemiluminescent substrate (50∶50 substrate and diluent) was added per well. Signals were captured and data were extracted and analysed as previously described [24].

#### 1.3 Data analyses

The 8 antibody response RLU-signals were blank-corrected by subtracting a blank spot signal specific to each sample. Initially, all zero or negative test-result values were converted to 0.0001 to allow for logarithmic transformation; however, the logarithmic transformation did not improve ROC curves. The blank-corrected values with negative values converted to 0.0001 will be referred to as “converted RLU”. Descriptive statistics (mean, standard deviation, minimum, maximum and median) of the converted RLU to the 8 antigens were calculated separately for non infected (NI) and infected (I) badgers. ROC curves for each antigen were constructed, and the ROC curve showing the largest sensitivity, with specificity set at 99.99%, is presented in this manuscript.

A stepwise logistic regression, with culture status as the dependent variable, the converted RLU to each of the 8 antigens as the independent variables, and using a significance level of 0.05, was carried out. Analytical weights were used to account for the cost of false positive test results being higher than that of negative test results. After exploring different cost ratios, a cost ratio of 100∶1 (false positive: false negative) was selected. Cost ratios higher than 100∶1 did not improve the ROC curve. From the logit obtained after using logistic regression, a cut off was chosen that allowed sensitivity to be maximised for specificity equal to 99.99%. A Hosmer-Lemeshow test was used to assess the goodness of fit of the final model. For the best linear combination of antigens, a ROC curve was created. All analyses were performed using Stata version 12 (Stata Corp, College Station, TX, USA).

### 2. Evaluation of test characteristics in vaccinated and non-vaccinated captive badgers

#### 2.1 Study 1 samples

Serum samples were available from a laboratory vaccine trial (Study 1). This trial was conducted to compare the levels of protection between vaccinated and non-vaccinated badgers, and between badgers vaccinated with different vaccines. Briefly, badgers were sourced from an area free of bTB and allowed to adapt to captivity for 12 weeks prior to the start of the experiment. During adaptation, the badgers were screened for tuberculosis using a lymphocyte transformation assay (LTA). The experiment consisted of three groups of badgers: animals vaccinated (10^8^ CFU) with either BCG Danish 1331 (n = 8 animals) or BCG Pasteur 1173P2 (n = 7), and controls (n = 8). All badgers were challenged by the endo-bronchial route with 6×10^3^ CFU *M. bovis*. The badgers were euthanised 15 weeks post-challenge and subjected to a detailed post-mortem examination. Blood samples were taken twice a month prior to vaccination (6 samples per badger) and once a month subsequently (2 samples prior and 4 samples subsequent to challenge, per badger).

#### 2.2 Study 2 samples

Serum samples were also available from a second captive badger study (Study 2). Data were available from a group of 9 badgers that were vaccinated (10^8^ CFU, BCG Danish strain) and a group of 10 badgers that served as a control group. Badgers were challenged with 3×10^2^ CFU of *M. bovis* by the endo-bronchial route, and followed for 51 weeks after challenge. Blood samples were taken every two weeks before badgers were vaccinated (3 samples per badger) and once a month subsequent to vaccination (3 samples before and 10 samples after challenge per badger) with a further sample taken before badgers were euthanized three months later.

#### 2.3 Multiplex immunoassay

The assay was conducted as described previously.

#### 2.4 Data analysis

For each of the captive studies, descriptive statistics of the antibody responses to each of the 8 antigens were calculated independently for each of the following badger categories: non-vaccinated – non-infected (Category 1), vaccinated – non-infected (Category 2), non-vaccinated - infected (Category 3) and vaccinated - infected (Category 4). In Study 1, the descriptive analysis was done taking into account only those badgers vaccinated with the Danish strain and then repeated using data from both groups of vaccinated badgers (Danish and Pasteur).

The optimal antigen combination (described in section 2.1) was applied to the data from Study 1 and Study 2 resulting in estimated logits; by applying the selected cut-off to the logits obtained, the sensitivity and specificity of the multiplex immunoassay test was estimated separately for each of the studies. In order to be consistent, only samples from badgers vaccinated with the Danish strain were used for estimating sensitivity and specificity in Study 1.The sensitivity and specificity was also estimated separately for vaccinated and non-vaccinated badgers in Study 2. Badgers in these datasets were considered infected one day after they had been challenged. For Study 2 data, the probability of testing positive was calculated from the logit using the formula: prob = exp(logit)/(1+exp(logit)). The cut-off value was also converted into a probability using the same formula. Scatter plots of the probability of testing positive by time since the start of the trial and least squares means of these probabilities were created separately for the control and vaccinated groups in Study 2. In order to explore whether the logit was associated with time since infection, a generalized estimating equations model (GEE; to account for repeated measures within a badger) with a vaccination-time interaction term was conducted. The model used a Gaussian distribution with identity link and exchangeable correlation structure. Quadratic and logarithmic transformations of the independent variable “time since challenge” were carried out but did not yield lower values of QIC (quasilikelihood under the independence model criterion). The working correlation for the repeated effect was 0.288. A two-way graph was created using the predictions of the GEE model and time since infection by vaccination status. All statistics were carried out using Stata version 12 (Stata Corp, College Station, TX, USA).

## Results

### 1. Test optimisation

In total, 78 of the 215 samples (36.3%) were infected with *M. bovis* based on culture results. Descriptive statistics for converted RLU response to each of the 8 antigens by infection status are presented in Table 1.

**Table 1:** Mean, standard deviation, maximum and median values for converted RLU response to each of the 8 antigens, by infection status (based on culture).

Antigen	Mean		SD		Max		Median	
	NI	I	NI	I	NI	I	NI	I
MPB70*	53.1	57.9	119.2	144.4	840.0	919.0	0.0	0.0
MPB70	161.1	4611.2	808.7	10845.5	9057.5	53843.0	6.5	106.3
MPB83	120.4	5081.5	365.3	11470.1	3160.0	53718.0	10.5	127.3
Rv3616c**	132.7	3627.7	498.3	9748.9	4952.0	53780.5	16.0	85.0
PPDb	124.7	3533.6	392.9	9205.1	2899.3	53749.3	15.5	74.6
Rv3616c	379.7	7303.4	1545.8	9205.1	13494.5	59739.0	7.5	263.8
ESAT-6	49.1	859.0	88.5	6866.2	494.5	60639.0	0.0	0.0
CFP-10	237.0	1501.6	924.4	7112.3	8605.5	53182.5	23.0	20.5

*MPB70 peptide,

**Rv3616c fragment.

**Figure 1:**
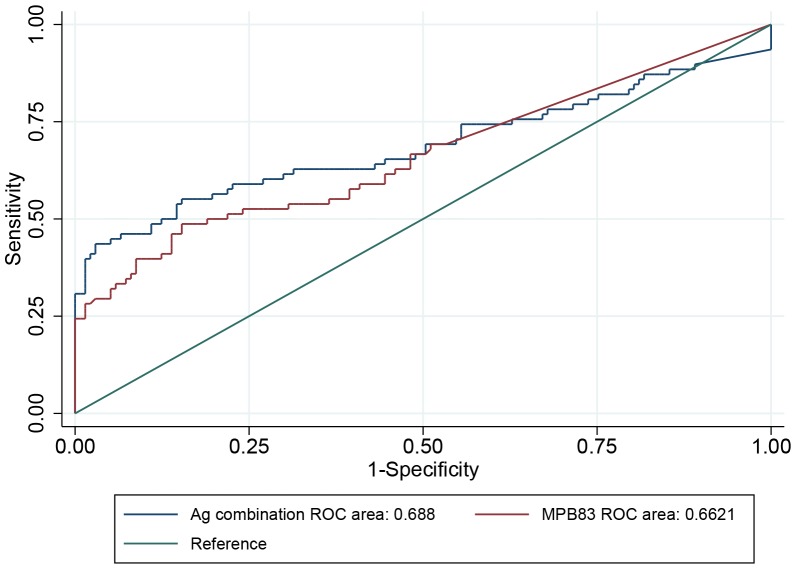
ROC curves of the logit obtained using either the optimised combination of antigens (blue line) or MPB83 (red line). Each is based on converted RLU values, and the green line is included for reference.

When using stepwise logistic regression with analytic weights, 7 of the 8 antigens were retained in the final model, but MPB70 peptide was not. The Hosmer-Lemeshow test showed that the model fitted the data sufficiently well (p-value 0.29). Figure 1 depicts the ROC curve of the logit obtained for the combination of antigens. For a specificity of 99.99%, a logit cut-off of −2.67 was needed. At this cut-off, the sensitivity was 30.77%. Of the single antigens, MPB83 achieved the largest sensitivity, 24.36%, with the specificity set at 99.99% (Figure 1).

### 2. Evaluation of test characteristics in vaccinated and non vaccinated badgers

In Study 1, 258 samples were analysed, with 30% of the badgers being tested 11 times and 70% of them 12 times. In Study 2, 297 samples were analysed (two samples could not be analysed for Rv3616c (fragment) due to insufficient serum, and were removed from the study), with a mean of 15.6 samples per badger (min = 9, max = 17). A table showing the descriptive statistics for each of the 8 antigens as converted RLU, by infection and vaccination status, is presented as (Table S1). This file also presents data for Study 1 samples originating from badgers vaccinated with the Danish strain.

Using the optimal antigen combination, the mean sensitivity and specificity of the multiplex immunoassay test were respectively 22.99% (CI:14.64–33.25%) and 78.95% (CI:72.07–84.80%) for Study 1, and 33.51% (CI:26.76–40.81%) and 83.04% (CI: 74.78%–89.47) for Study 2. The sensitivity and specificity were also calculated separately for vaccinated and non-vaccinated badgers from Study 2, obtaining sensitivity values for vaccinated badgers of 32.26 (CI:22.93–42.75%) and specificity of 88.89% (CI:70.84–97.65%); the equivalent values for non-vaccinated badgers were 34.78% (CI:25.15–45.43%) sensitivity and 81.18% (CI:71.24–88.84%) specificity.

**Figure 2:**
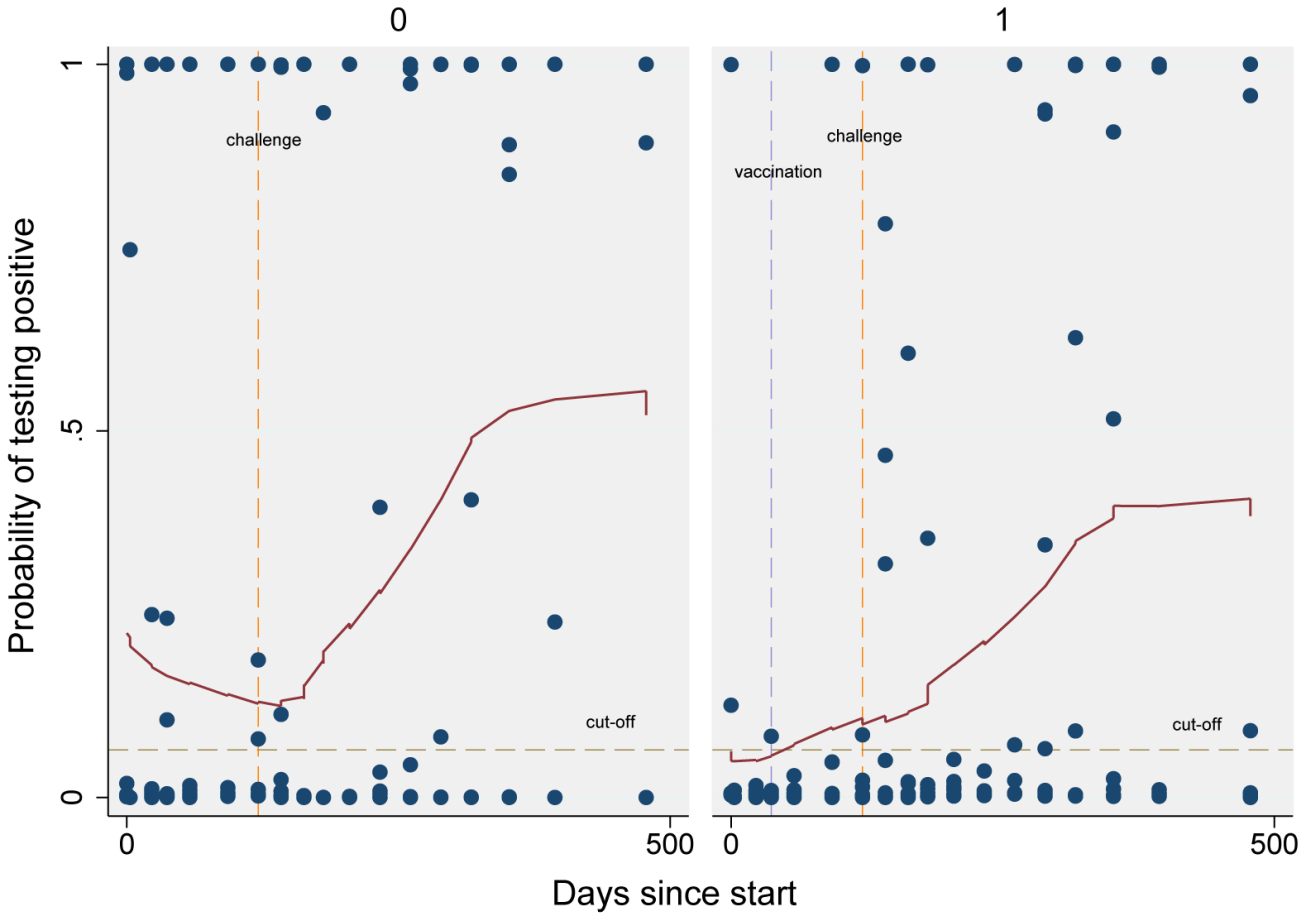
Scatter plot and least square means of the probability of testing positive for the control (left graph) and vaccinated (right) groups in Study 2 by time since the start of the study. Vertical reference lines showing the day of vaccination and challenge, and a horizontal reference line of the cut-off are included.

Figure 2 presents scatter plots and least square means of the probability of testing positive for the control and vaccinated groups in Study 2 by time since the start of the study.

**Figure 3:**
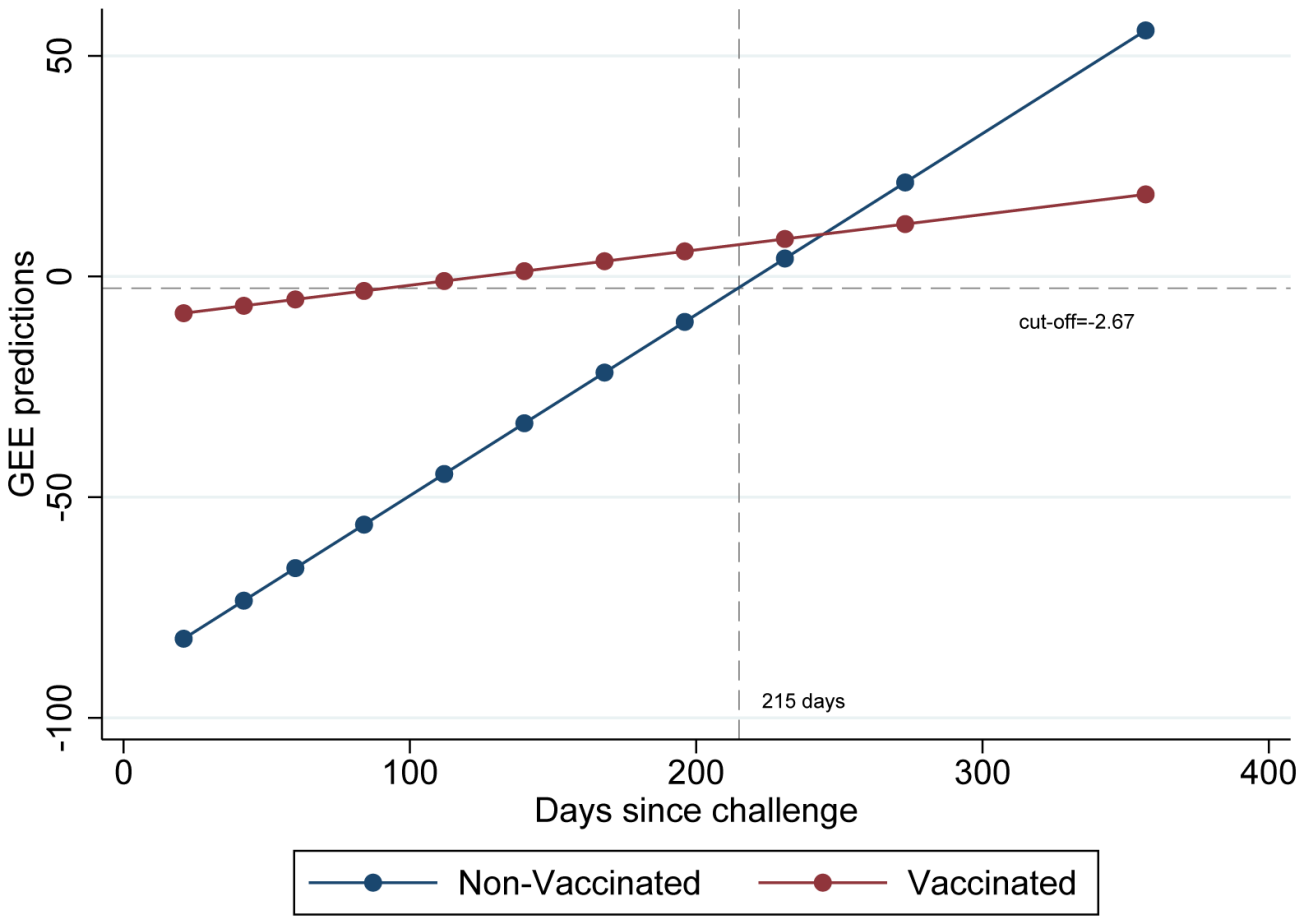
GEE predictions by time since challenge for vaccinated and non-vaccinated badgers. Two reference lines are presented: a vertical line showing the minimum number of days to test positive for infected non-vaccinated badgers and a horizontal cut-off line.

A two-way graph showing the GEE predictions by time since challenge is presented in Figure 3. In this Figure, the rate of increase of the logit is presented separately for vaccinated and non-vaccinated badgers. The GEE analysis showed a significant association of the dependent variable with all three independent variables: time since challenge, vaccination and their interaction term (p<0.005). When a robust GEE model was fitted, the interaction term was borderline significant (p = 0.045). In the Figure, a line showing the selected cut off value is highlighted. A reference line showing the minimum number of days to test positive for infected non-vaccinated badgers is also presented.

## Discussion

The main objective of this paper was to optimise a diagnostic test for *M. bovis* infection in live badgers, for use during the evaluation of vaccine efficacy in a large bTB vaccine field trial in Ireland. On most occasions when a test is being developed, it is desirable to optimise both the sensitivity and the specificity of the test; in such situations, a cost ratio for false positives and false negatives of 1∶1 is selected. However, in a previous study focusing on the statistical power of this trial [11], it was demonstrated that the diagnostic test needed to be tailored to achieve a specificity close to 100% to achieve a reasonable statistical power (60–80%). As the incidence of *M. bovis* infection in the badger population decreases, there will be an increasing number of false positive results, randomly occurring in the mainly negative samples from both vaccinated and unvaccinated animals, with the potential to greatly bias estimates of vaccine efficacy. Hence, the need for specificity close to 100%, thereby minimising the number of false positive results. Greiner et al. (2000) [27] have shown that analytic weights can be used to optimise cut-off values with regard to a specific cost ratio of false positive and false negative results. In order to reduce the number of false positive results, the upper left corner of the ROC curve (draw as sensitivity against 1-specificity) was optimised by selecting a cost ratio of false positives to false negatives equal to 100∶1. Other ratios were explored, noting that cost ratios higher than 100∶1 did not improve the ROC curve, probably due to the relatively small number of samples available to test. Subsequently, a cut-off value of a logit equal to −2.67 was needed to achieve a specificity of 99.99%, resulting in a maximum sensitivity of 30.77%. This sensitivity, although quite low in comparison to generally available diagnostic tests, is sufficient to achieve a statistical power of the vaccine trial of over 80% [11]. In this trial, test sensitivity is of lesser importance on study power because, when the incidence of *M. bovis* infection in badgers is low, the expected proportion of false negatives will be a fraction of something that is already a very small proportion.

In this study, culture was used to ascertain the disease status of individual badgers. Although it is unlikely that culture is a perfect ‘gold standard’, the methodology used in this study is based on an enhanced post mortem technique, currently the most sensitive available. In recent years, a progressive increase in estimated prevalence of *M. bovis* infection in badgers has been observed, both in Ireland and the UK, attributable to improved sensitivity of detection [3], [4]. Crawshaw et al. (2008) [28] reported a 54% sensitivity of a standard post-mortem procedure compared to a more detailed enhanced post mortem technique.

A second objective in this paper was to assess the effects of vaccination on test characteristics. The optimised test described above was applied to data from Study 1 and 2. Badgers in Study 2 were followed for a much longer period of time subsequent to challenge than badgers in Study 1. Therefore data from Study 2, as opposed to Study 1, were used for further analysis. The sensitivities/specificities for non- vaccinated and vaccinated badgers in Study 2 were 34.78/77.97% and 32.26/88.68%, respectively. The test characteristics for both vaccinated and non-vaccinated badgers were very similar, as indicated by the overlap of the confidence intervals. Nonetheless, the specificity obtained for Study 2 was lower than that obtained when the test was optimised in naturally infected individuals (99.99%). When looking at the specific badger data presented in Figure 2, it was observed that a large proportion of the false positives samples within the control group badgers belonged to two badgers that repeatedly tested positive prior to challenge (6 and 4 times per badger respectively, out of a total of 6 sampling times during this period). One possibility is that those two badgers were infected prior to the start of the experiment. However, blood samples from these animals were repeatedly screened before challenge by the more sensitive lymphocyte transformation assay using bovine and avian tuberculins, and were negative on all occasions (data not shown). The underlying cause of the false positive reactions in the two badgers is unknown though cross reactivity can potentially arise from concurrent infections with related pathogens or any microbe with shared epitopes, all of which potentially influence the specificity of this test. By removing samples from those two badgers, the specificity of the test increased to 93.62% in non-vaccinated badgers and to 91.00% for all badgers (vaccinated and non-vaccinated combined). Contrary to the control group, the false positives samples observed in the vaccinated group belonged to different badgers. These badgers tested positive (prior to challenge) on no more than one occasion, out of an average of 6 tests per badger during this period.

One of the concerns of serologically-based assays is that they detect infection later than assays based on the cell-mediated immune response, such as gamma-interferon. This is because the initial immune response is cell-mediated. A lag between infection and positive test results has been observed in the data obtained from Study 2 (Figures 2 and 3); this lag varies between vaccinated and non-vaccinated animals. From a biological point of view, we would expect that non-vaccinated badgers will carry a larger antigen load and thus will mount a larger antibody response to *M. bovis* challenge than those that are vaccinated. Nonetheless, it is the vaccinated group that shows the earliest positive test when a cut-off = −2.67 is selected (Figure 3 shows samples of vaccinated badgers testing positive earlier than 100 days after challenge, while the equivalent for the non-vaccinated group was 215 days). We can think of two possible interpretations for the observed results, one is that vaccinated badgers will mount a serological response faster than non-vaccinated badgers following infection, the other option is that observed results are due to chance (due to the small number of badgers in each group). It is possible that some of the badgers that were randomly allocated to the vaccinated group were extremely susceptible to infection and for those badgers, vaccination did not work.

Considering all of the above, what we propose in this study is that the multiplex immunoassay can be used to analyse the vaccine trial data, incorporating the optimal antigen combination identified from section 2.1 and a consistent cut-off of -2.67. To account for the differences observed in the lag between time of infection and a positive test, we recommend that only subsequent captures that occur more than “Y” days apart are used for the analysis, with “Y” being the minimum number of days necessary between infection and a subsequent positive test (215 days in this study). The number “Y” can be determined, after the vaccine trial dataset is gathered, as a trade-off between the increase in sensitivity and the possible reduction in power resulting from a decrease of our sample size. By taking this approach, it will be possible to minimise bias, specifically the incorrect classification of infected animals as non-infected.

In summary, a multi-antigen test has been optimised for use during the evaluation of vaccine effectiveness in a badger bTB vaccine field trial in Ireland. During optimisation, test sensitivity was estimated, while specificity was set at 99.99%. Based on the operating characteristics of the diagnostic test, it has been demonstrated that the statistical power of the field trial could exceed 80% [11]. We have also observed that test characteristics do not vary greatly between vaccinated and non-vaccinated badgers. In relation to the time lag between infection and a positive test in vaccinated and non-vaccinated badgers, we have presented a strategy to enable the test to be used, and applied consistently, during trial evaluation.

## Supporting Information

Table S1 **Mean, standard deviation, maximum and median values for converted RLU response to each of the 8 antigens, by infection and vaccination status (Categories 1 to 4).**
(XLS)
